# Acknowledgment to reviewers of *Extracellular Vesicles and Circulating Nucleic Acids* in 2023

**DOI:** 10.20517/evcna.2024.01

**Published:** 2024-01-12

**Authors:** EVCNA Editorial Office

**Affiliations:** Suite 1504, Tower A, Xi’an National Digital Publishing Base, No. 996 Tiangu 7th Road, Gaoxin District, Xi’an 710077, Shaanxi, China.

The Editorial Office of *EVCNA* would like to extend its sincere gratitude and appreciation to the following reviewers for dedicating their valuable time and efforts to evaluating the received manuscripts throughout 2023, regardless of whether the papers they reviewed were finally published [Table 1].

**Table 1:** Reviewers in 2023

Abhigyan Satyam (U.S.)	Hua Wang (China)	Paolo Paganetti (Switzerland)
Ali Syed Arbab (U.S.)	Huantian Zhang (China)	Peter Celec (Slovakia)
Andrew Ewing (Sweden)	Illimar Altosaar (Canada)	Phil G. Campbell (U.S.)
Anne-Mari Mustonen (Finland)	Inge S. Zuhorn (Netherland)	Pidder Jansen-Duerr (Australia)
Antonio Marcilla (Spain)	Isaac Kirubakaran Sundar (U.S.)	Pietro Parisse (Italy)
Aria Baniahmad (Germany)	Italia Di Liegro (Italy)	Pinar Uysal-Onganer (U.K.)
Arianna Scuteri (Italy)	Janusz W. Rak (Canada)	Pranav Sharma (U.S.)
Ashley E. Russell (U.S.)	Javier Vaquero (Spain)	Qiong Liu (China)
Aurélie Ledreux (U.S.)	Jeremy A Garson (U.K.)	R. J. Griffin (U.S.)
Barbara Slusher (U.S.)	Jiacan Su (China)	Ramaroson Andriantsitohaina (France)
Beth Lee (U.S.)	Jiali Tan (China)	Ramin M. Hakami (U.S.)
Bing Guo (China)	Jiujiu Yu (U.S.)	Regina Golan-Gerstl (Israel)
Bing-Dong Sui (China)	Joan Oliva (U.S.)	Renata Del Carratore (Italy)
Brian Sims (U.S.)	Joerg Bredno (U.S.)	Ricardo J. Ferreira (Sweden)
Camino de Juan (Spain)	John Williams III (U.S.)	Richard J. Simpson (Australia)
Carine De Marcos Lousa (U.K.)	Jonghoon Choi (Korea)	Robert Eibl (Switzerland)
Carl Randall Harrell (U.S.)	Juan Antonio Fafián Labora (Spain)	Robert L. Raffaï (U.S.)
Carolina Balbi (Switzerland)	Juan Du (China)	Roberto Cannataro (Italy)
Celeste Caruso Bavisotto (Italy)	Julien Villeneuve (France)	Sabrina Giglio (Italy)
Chao Wang (China)	Juliette Nectoux (France)	Seiko Ikezu (U.S.)
Chen-Huan Yu (China)	Junko Fujihara (Japan)	Sonia Alonso-Martin (Spain)
Chi Wut Wong (U.S.)	Justyna Karkowska-Kuleta (Poland)	Ssang-goo Cho (Korea)
Chiara Fenoglio (Italy)	Karl-Heinz Kogel (Germany)	Stelvio Tonello (Italy)
Christina Coughlan (U.S.)	Ki Soo Park (Korea)	Stephen F. Badylak (U.S.)
Christopher D. Gregory (U.K.)	Kiyotaka Shiba (Japan)	Subhadip Ghatak (U.S.)
Claudia Goettsch (Germany)	Kohei Yuyama (Japan)	Su-Hua Qi (China)
Claudia Verderio (Italy)	Koji Ueda (Japan)	Suman Dutta (U.K.)
Consuelo Borrás (Spain)	Kotb Abdelmohsen (U.S.)	Susanne Gabrielsson (Sweden)
Crislyn D'Souza-Schorey (U.S.)	Kyungmoo Yea (Korea)	Takao Yasui (Japan)
Daniel Piotr Potaczek (Germany)	Lang Rao (China)	Tatiana Y Kostrominova (U.S.)
David M. Lubman (U.S.)	Lexie Shannon Holliday (U.S.)	Theresa L. Whiteside (U.S.)
Davide Chiasserini (Italy)	Lianmei Zhao (China)	Thomas F Martin (U.S.)
Dongin Kim (U.S.)	Lorena Urbanelli (Italy)	Tianyu Zhong (China)
Edward M. Campbell (U.S.)	Luana Lugini (Italy)	Valentina Bollati (Italy)
Emily Sims (U.S.)	Lucia R. Languino (U.S.)	Vito G. D'Agostino (Italy)
Enrico Ragni (Italy)	Lynne Bemis (U.S.)	Wang-ngai Chow (China)
Erika Cione (Italy)	Maija Puhka (Finland)	Wei Seong Toh (Singapore)
Eva-Maria Krämer-Albers (Germany)	Marcelo A. Lima (U.K.)	Wenwan Zhong (U.S.)
Fabrice Lucien (U.S.)	Maria Carmen Blanco-López (Spain)	William A. Banks (U.S.)
Farah Asa'ad (Sweden)	Maria Felice Brizzi (Italy)	William Duddy (U.K.)
Fernando De la Cuesta (Spain)	Maria Pia Foschino Barbaro (Italy)	Xi Wei (China)
Fusheng Quan (China)	Marina Leite (Portugal)	Xing Li (China)
Gagan Deep (U.S.)	Mathew Wood (U.K.)	Yadong Zheng (China)
Gal Bitan (U.S.)	Michael Bukrinsky (U.S.)	Yang You (U.S.)
Geneviève Bart (Finland)	Michela Relucenti (Italy)	Yaohui Tang (China)
George A Calin (U.S.)	Morvane Colin (France)	Yazhen Zhu (U.S.)
Gregor Eichele (Germany)	Murali Yallapu (U.S.)	Yong Chen (China)
Grégory Lavieu (France)	Murray David M. Mitchell (Australia)	Yong-Liang Zhang (China)
Guoku Hu (U.S.)	Natalie Chandler (U.K.)	Young-Eun Cho (Korea)
Guoping Li (U.S.)	Naureen Javeed (U.S.)	Yusuke Yoshioka (Japan)
Guozhen Liu (China)	Nicole Noren Hooten (U.S.)	Yuxia Luan (China)
Hans Thordal-Christensen (Denmark)	O. Trubiani (Italy)	Zhaohui Huang (China)
Hargita Hegyesi (Hungary)	Olga M. Shatnyeva (Sweden)	Zhaowei Chen (China)
Hilde Kanli Galtung (Norway)	Orazio Fortunato (Italy)	Zhijian Cai (China)
Hongchen Sun (China)	Paola Margutti (Italy)	Zhijin Fan (China)

The invaluable contributions of our expert reviewers play a pivotal role in the journal’s editorial decision-making process. In gratitude for our reviewers, we will create individual accounts for them in the submission system in 2024. This will allow them to access details of their past reviews, view comments from other reviewers, and download letters of acknowledgment for their records. As a further token of appreciation, the Editorial Office will select two outstanding individuals as the “Best Reviewers” in 2024, and each awardee will be awarded USD 350. Once again, we sincerely thank all our reviewers for their generosity and commitment in the previous year.

## DECLARATIONS

### Copyright

©The Author(s) 2024.

